# Identification of Host Defense-Related Proteins Using Label-Free Quantitative Proteomic Analysis of Milk Whey from Cows with *Staphylococcus aureus* Subclinical Mastitis

**DOI:** 10.3390/ijms19010078

**Published:** 2017-12-28

**Authors:** Shaimaa Abdelmegid, Jayaseelan Murugaiyan, Mohamed Abo-Ismail, Jeff L. Caswell, David Kelton, Gordon M. Kirby

**Affiliations:** 1 Department of Biomedical Sciences, Ontario Veterinary College, University of Guelph, Guelph, ON N1G 2W1, Canada; sabdelme@uoguelph.ca; 2 Institute of Animal Hygiene and Environmental Health, Centre for Infectious Medicine, Freie Universität Berlin, 14163 Berlin, Germany; 3 Department of Animal Biosciences, Ontario Agriculture College, University of Guelph, Guelph, ON N1G 2W1, Canada; maboisma@uoguelph.ca; 4 Department of Pathobiology, Ontario Veterinary College, University of Guelph, Guelph, ON N1G 2W1, Canada; jcaswell@uoguelph.ca; 5 Department of Population Medicine, Ontario Veterinary College, University of Guelph, Guelph, ON N1G 2W1, Canada; dkelton@uoguelph.ca

**Keywords:** mastitis, bovine, milk proteomics, whey, milk proteins, *Staphylococcus aureus*

## Abstract

*Staphylococcus aureus* is the most common contagious pathogen associated with bovine subclinical mastitis. Current diagnosis of *S. aureus* mastitis is based on bacteriological culture of milk samples and somatic cell counts, which lack either sensitivity or specificity. Identification of milk proteins that contribute to host defense and their variable responses to pathogenic stimuli would enable the characterization of putative biomarkers of subclinical mastitis. To accomplish this, milk whey samples from healthy and mastitic dairy cows were analyzed using a label-free quantitative proteomics approach. In total, 90 proteins were identified, of which 25 showed significant differential abundance between healthy and mastitic samples. In silico functional analyses indicated the involvement of the differentially abundant proteins in biological mechanisms and signaling pathways related to host defense including pathogen-recognition, direct antimicrobial function, and the acute-phase response. This proteomics and bioinformatics analysis not only facilitates the identification of putative biomarkers of *S. aureus* subclinical mastitis but also recapitulates previous findings demonstrating the abundance of host defense proteins in intramammary infection. All mass spectrometry data are available via ProteomeXchange with identifier *PXD007516*.

## 1. Introduction

Bovine mastitis is most often caused by a bacterial infection and results in severe losses in milk production and in the deterioration of the physical and chemical composition of the milk [1,2]. Mastitis is estimated to cost the Canadian dairy industry more than $300 million annually [3]. This has a huge economic impact on the dairy industry as well as concerns of zoonotic disease transmission and contributions to the emergence of antimicrobial resistance [4]. Clinical mastitis is usually associated with visible local and systemic signs of inflammation of the udder and with abnormalities in the secreted milk such as watery consistency, milk clots, flakes, or evidence of blood or pus. In contrast, subclinical mastitis (SCM) lacks recognizable inflammatory manifestations, so the infected animals can go undetected, and the infection might advance to a chronic stage resulting in unresponsiveness to treatment. *Staphylococcus aureus* is one of the most frequently isolated bacterial pathogens from persistent subclinical mastitis cases [1,4]. In Canada, herd level prevalence ranges from 40% in British Columbia, 60% in Alberta, ~70% in Ontario and Quebec, and up to 90% in Saskatchewan and Nova Scotia [5]. Currently, there are only limited diagnostic tests performed on milk to detect subclinical intramammary infection (IMI) due to *S. aureus*, and those that are available have moderate specificity and sensitivity [6,7]. Therefore, there is a critical need to develop tests that detect the early stage of subclinical mastitis, allowing for efficient treatment of diseased cows and thereby minimizing the spread of the infection throughout the herd.

Host–pathogen interactions are variable during the different stages of mastitis and are important determinants of the outcome of the infection [8,9]. Indeed, the outcome—either elimination of the pathogen from the mammary glands or establishment of the infection—is strongly dependent on the host immune response [10,11]. If bacteria gain access to mammary tissues by evading the teat anatomical barriers, a typical first line of host defense is the activation of the innate immune response either by recognition of pathogen molecules or by upregulation of the local resident immune components within the mammary tissues. Changes in the abundance levels of innate immune mediators and modulation in their abundance patterns could act as biomarkers of the host response to predict early intramammary infection with specific pathogens [12,13,14].

Recent advances in proteomic technologies, together with rapidly evolving bioinformatics tools, allows for in-depth analyses of different fractions of the milk proteome, especially the low-abundant proteins [15,16,17]. Accordingly, the aim of this study was to profile and quantify the differential abundance of milk whey proteins in cows with subclinical mastitis naturally infected with *S. aureus* compared with that in healthy control animals. *S. aureus* was chosen in order to reduce confounding factors that might arise as a result of examination of subclinical mastitis due to a variety of different pathogens. In the present study, a proteomic approach involving liquid chromatography and tandem mass spectrometry was applied to whey samples to identify potential biomarker proteins of subclinical mastitis. Furthermore, the biological relevance of the differentially abundant proteins in subclinical mastitis was investigated using various bioinformatics strategies to connect proteins to known biological functions and pathways.

## 2. Results

**Figure 1:**
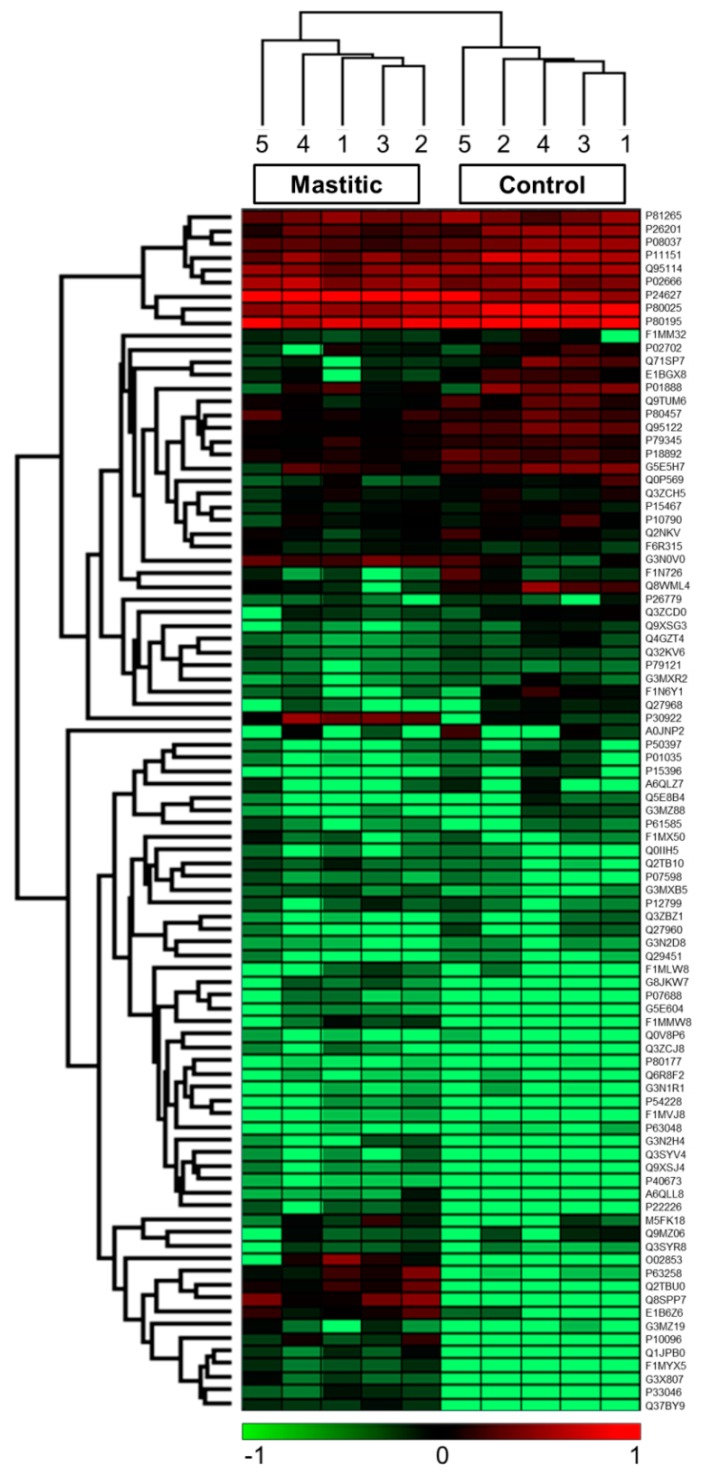
Heat map representing the hierarchical clustering analysis of the identified proteins and the proteins that were significantly high-/low-abundant in the mastitic group compared with the control group. Data analysis was based on label-free quantification (LFQ) and was performed in Perseus software using five biological replicates of both control and mastitic groups. Columns represent samples; rows are individual proteins. Red indicates high-abundant proteins and green indicates low-abundant proteins. UniProt ID numbers are indicated on the right.

Shotgun proteomic analyses of bovine milk whey proteins involving trypsin digestion, separation of peptides by liquid chromatography (LC) coupled to MS/MS, and label-free proteomic analyses revealed modulation of the milk proteome in cows infected with *S. aureus* and differential abundance of proteins with host defense functions. A MaxQuant software-based search against the *Bos taurus* proteome database resulted in the identification of 90 proteins (Table S1), among which 25 proteins were differentially abundant between the control and mastitic groups. Fifteen proteins were identified with high abundance, whereas ten were identified with low abundance in comparison with the control group Table 1. The hierarchical clustering analysis of the identified proteins (Figure 1) revealed two different clusters—one each for control and mastitis samples. One of the diseased samples (D6) was excluded from the clustering analysis due to a weak peptides signal compared with the other replicates.

Gene Ontology (GO) enrichment analysis using Database for Annotation, Visualization, and Integrated Discovery (DAVID) software revealed several categories related to host defense, the most relevant of which are as follows: 29.1% of identified proteins were associated with “response to bacterium” (GO:0009617), 25% with “regulation of cytokine production” (GO:0001817), 20.8% with the “innate immune response” (GO:0045087), 12.5% with the “toll-like receptor signaling pathway” (GO:0002224), and 8.3% with “cellular response to lipoteichoic acid” (GO:0071223). The complete list of the GO terms that were enriched in the analysis is provided in Table S2. The most relevant GO biological processes in which the proteins were annotated are shown in Table 2. The direct GO option of DAVID was used at *p* < 0.05.

**Figure 2:**
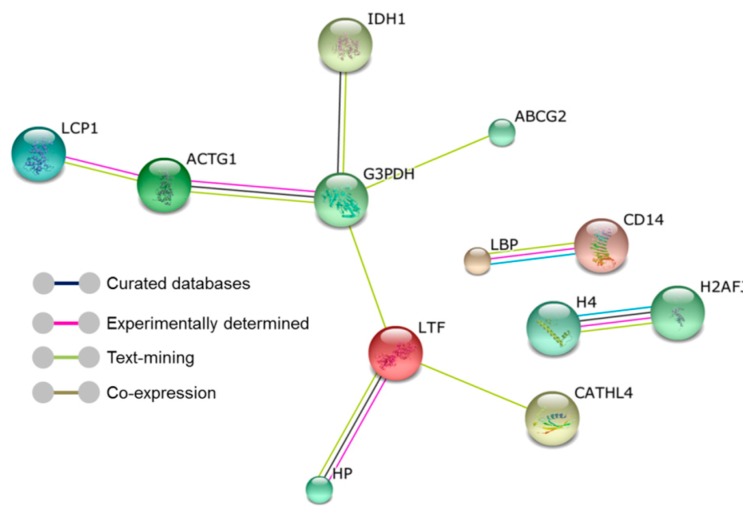
The protein–protein interaction network analysis using Search Tool for the Retrieval of Interacting Genes/Proteins (STRING) software. The nodes represent individual proteins enriched in the analysis and the edges (connecting lines) reflect the functional associations derived from various online resources. Protein nodes which are enlarged indicate the availability of 3D protein structure information. LBP = Lipopolysaccharide-binding protein, CD14 = Monocyte differentiation antigen, H4 = Histone, H2AFJ = Histone H2A, LTF = Lactotransferrin, CATHL4 = Cathelicidin-4, HP = Haptoglobin, IDH1 = Isocitrate dehydrogenase, ABCG2 = ATP-binding cassette subfamily G member 2, GAPDH = Glyceraldehyde-3-phosphate dehydrogenase, ACTG1 = Actin cytoplasmic 2, and LCP1 = Uncharacterized protein.

Protein–protein interaction (PPI) was investigated using the Search Tool for the Retrieval of Interacting Genes/Proteins (STRING) database. Twelve proteins were enriched in networks, each one representing nodes of which nine proteins showed interactions reflected by connecting lines or edges (Figure 2). The confidence score of the interaction networks ranged from 0.526 to 0.951. The higher confidence score reflects the higher probability that the interaction is biologically meaningful given that it is supported by several types of evidence such as curated databases, or was experimentally determined.

**Figure 3:**
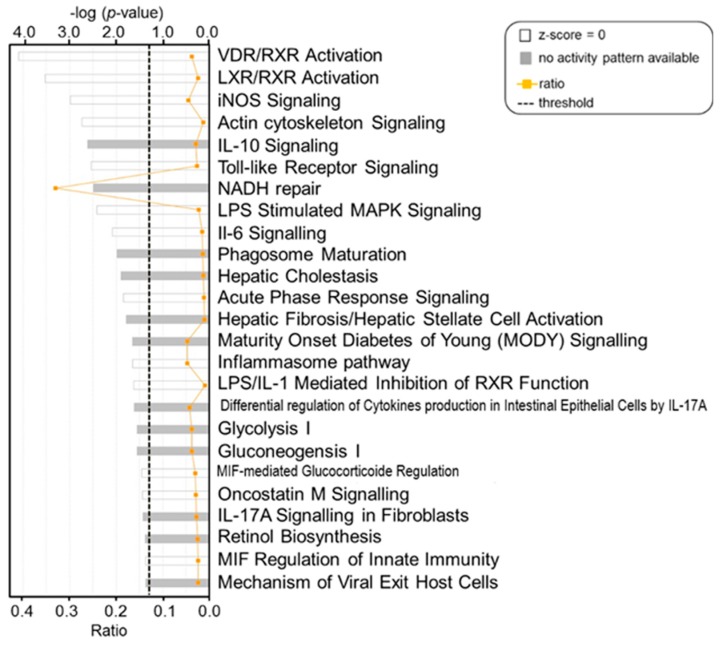
Ingenuity-based identification of key signaling canonical pathways enriched in proteins in milk infected with *S. aureus*. Each pathway is represented with a bar, the length of which shows the negative log of the *p*-value obtained by a Right Tailed Fisher’s Exact Test (the longer the bar, the more significant the enrichment) and the color reflects available information on the activity pattern (i.e., protein abundance). White bars: *Z*-score = 0 indicates protein abundance is enriched in the pathway (i.e., either high-abundant or low-abundant); grey bars: indicate that no activity pattern is available in the software database. Ratio indicates the proportion of differentially abundant proteins found in the current dataset relative to the total number of proteins contributing to that pathway (e.g., a ratio of 0.2 indicates that 20% of the proteins in the pathway were identified in the dataset). VDR/RXR = Vitamin D receptor, RXR = retinoid X receptor, LXR = liver X receptor, RXR = retinoid X receptor, NOS = nitric oxide synthase, IL = interleukin.

Twenty-three differentially abundant proteins were found to be enriched in most of the Canonical pathways and network analyses performed using Ingenuity *Pathway* Analysis (IPA). As shown in Figure 3, many of the enriched pathways reflect the involvement of proteins with host defense functions such as the Toll-like Receptor Signaling pathway in which lipopolysaccharide-binding protein (LBP) and monocyte differentiation antigen (CD14) were enriched. Similarly, haptoglobin (Hp) and lactotransferrin (LTF) were enriched in the acute-phase response. Additionally, the differentially abundant proteins in our dataset were enriched in vitamin D receptor/ retinoid X receptor (VDR/RXR) activation, liver X receptor/retinoid X receptor (LXR/RXR) activation, inducible nitric oxide synthase (iNOS) signaling, interleukin-6 (IL-6) signaling and acute-phase response signaling.

## 3. Discussion

The objective of the present study was to characterize the changes of the bovine milk whey proteome during *S. aureus* subclinical mastitis and to identify proteins that represent potential candidate biomarkers. The long-term goal is to validate key biomarker proteins in future studies and to eventually develop a diagnostic tool with improved sensitivity and specificity for subclinical mastitis. The LC/MS-based label-free quantitative proteomic analysis of milk whey samples from healthy and mastitic animals revealed a significant overabundance of proteins in mastitic milk. Of the 90 proteins identified, 25 are involved in well-characterized host defense functions or inflammatory processes. This suggests that infection with *S. aureus* is associated with a host immune response that is reflected at the level of the milk proteome. In contrast to other studies [16,17,18] that characterized altered protein levels during experimental *S. aureus* mastitis, our study investigated these alterations in samples collected from cows naturally infected with *S. aureus*. Collecting field data from naturally infected cows has the advantage of avoiding experimental design difficulties in terms of challenge levels and exposure probabilities; however, cows may be represented at various stages of progression of subclinical intramammary infection [19]. Furthermore, in the present study, preparation of milk samples by initial skimming and low speed centrifugation may have removed some proteins that might have been identified by other studies where pretreatment of milk samples was less extensive.

Identification of protein repertoires in the milk of cows affected by spontaneous mastitis is an appropriate and valuable strategy for discovery of potential biomarkers to be used in novel diagnostic tests with increased accuracy for subclinical mastitis. Bioinformatics analysis revealed that several differentially abundant proteins listed in Table 2 can be divided into subcategories of host defense biological functions. Haptoglobin (Hp) and lipopolysaccharide-binding protein (LBP) function as acute-phase proteins, while cathelicidin-4 (CATHL4), peptidoglycan recognition protein1 (PGLYRP1), lactoperoxidase (LPO), lactotransferrin (Lactoferrin) (LTF), histone proteins (histone H4 and Histone H2A), and cathepsin B (CTSB) have known antimicrobial activity. A third group functions as pathogen-recognition proteins including lipopolysaccharide-binding protein (LBP), monocyte differentiation antigen (CD14), and chitinase-3-like protein 1 (CHI3L1). Other differentially abundant proteins have known metabolic or structural functions such as lipoprotein lipase (LPL), beta-1,4-galactosyltransferase 1 (B4GALT1), glyceraldehyde-3-phosphate dehydrogenase (GAPDH), nucleobindin-1 (NUCB1), and Actin (ACTG). In the mammary glands, the immune response to different pathogens, including *S. aureus*, is a reflection of the inherent capability of the innate immune system to induce an integrative reaction to recognize, minimize, and overcome intramammary infection. This response starts with recognizing the highly conserved motifs which are known as pathogen-associated molecular patterns (PAMPs), such as lipoteichoic acid (LTA), peptidoglycan (PGN), or lipopolysaccharide (LPS). These are major components of the outer cell wall of gram-positive and gram-negative bacteria, respectively [11,20,21]. The recognition of PAMPs occurs through pattern recognition receptors (PRRs) which include the family of Toll-like receptors (TLRs) that can recognize specific PAMPs. Additionally, LBP also plays an important role in recognizing bacterial LPS and LTA during *E. coli* and *S. aureus* mastitis [11,22]. LBP delivers and facilitates the binding of bacterial LPS or PGN to membrane-bound CD14 (mCD14) or soluble CD14 (sCD14) to form a LBP–LPS–mCD14 complex. This complex is then recognized by TLR4 to induce an innate immune response to infection initiated by LPS or gram-negative bacteria. However, interaction of LBP with LTA or PGN of gram-positive bacteria and subsequent binding to mCD14 triggers a different innate immune response via TLR2 activation [23,24]. Results from our PPI network analysis highlighted the functional interaction between LBP and CD14 with a high confidence score (0.9). Moreover, GO analysis highlighted their involvement in biological processes relevant to the innate immune response such as “cellular response to LTA” (GO:0071223), “pattern recognition receptor signaling pathway” (GO:0002221), and “positive regulation of cytokine production” (GO:0001819). Therefore, the observation that LBP was high-abundant in this study is consistent with its known immune activities as one of the key host defense mechanisms that control intramammary infection. However, we found that that CD14 was low-abundant, which is in contrast to a previous study in mastitic cows which reported elevated levels of CD14 either in serum or milk whey following intramammary infection with *S. aureus* [20]. Nonetheless, our observation is in agreement with another study which investigated the levels of CD14 in *S. aureus* or *S. uberis* bovine mastitic milk postpartum and concluded that low levels of CD14-positive cells might be an indicator of mastitis following calving [25]. LBP protein also plays an important role as a major positive acute-phase protein which is synthesized in the liver and released into the general circulation and is produced locally in the mammary glands in response to the induction of pro-inflammatory cytokines such as IL-1 and IL-6 [26,27]. LBP was one of the proteins identified in the IL-6 signaling pathway in the Ingenuity pathway analysis.

Haptoglobin (HP) is another positive acute-phase protein that was high-abundant in mastitic milk in our study. Several studies demonstrated that Hp is a sensitive marker of mastitis, especially when measured in milk, rather than in serum, with increases in excess of 100-fold during earlier stages of intramammary infection (IMI) [28,29]. Moreover, Hp was high-abundant during acute mastitis caused by three different strains of *S. aureus* [17] or *E. coli* [26], suggesting that Hp is a sensitive nonspecific indicator of inflammation as it cannot discriminate between different pathogens. The main biological role of Hp is to bind to free hemoglobin-derived iron to render it unavailable to bacteria, resulting in a bacteriostatic effect, reducing bacterial growth and multiplication [22,29,30]. Following IMI, macrophages produce pro-inflammatory cytokines—predominantly IL-6, IL-1, and tumor necrosis factor alpha (TNFα)—that elicit an acute-phase response which stimulates the synthesis of Hp in the liver and locally in the mammary gland tissues. The other possible sources of Hp in milk during mastitis are somatic cells, predominantly neutrophils, or via leakage through the damaged blood–milk barrier [29,31]. Enrichment of Hp in the IL-6 signaling pathway in the IPA analysis in our dataset is consistent with the role that IL-6 plays in activation of the acute-phase response during inflammation, as was shown in previous studies.

Another group of host defense-related proteins identified in our study are diverse proteins with direct antimicrobial functions, produced by neutrophils and macrophages, that infiltrate the mammary glands in response to IMI or are synthesized locally by mammary epithelial cells [11,32,33,34]. Among this group of proteins, LTF, CATHL4, PGLYRP1, CTSB, H4, and H2A were high-abundant; however, LPO was low-abundant in mastitic whey milk compared with that of controls. LTF is recognized as an antimicrobial and bactericidal protein through its capacity to sequester Fe which deprives the bacteria of this critical element required for growth and replication. The levels and patterns of the LTF protein during clinical or subclinical mastitis were investigated in earlier studies that found LTF to be significantly increased in milk from quarters infected with *S. aureus* or *Streptococcus agalactiae* [35,36]. Similarly, another study suggested that levels of LTF protein are pathogen-specific as a significant abundance of LTF occurred during *S. uberis*, whereas *S. aureus* and *E. coli* IMI induced low levels of LTF during IMI [35]. LTF was identified and validated in a recent study as a putative diagnostic and prognostic biomarker for cows with high resistance to mastitis and other metabolic diseases versus low-resistance cows [37]. GO analysis showed enrichment of LTF in relevant biological processes such as “antibacterial humoral response” (GO:0019731) and “positive regulation of innate immune response” (GO:0045089). Moreover, the protein–protein interaction network analysis in the present study showed the well-known functional interaction between Hp and LTF in chelating free iron, which affects the growth of many pathogens.

CATHL4 is a member of ten cathelicidin genes that have previously been identified in bovine milk which are produced or secreted from neutrophil cytoplasmic granules and have well-characterized potent antimicrobial functions and proinflammatory activities [38,39]. Our results indicating high-abundance of CATHL4 in milk from cows infected with *S. aureus* subclinical mastitis concur with other proteomic studies [13,15,16,40]. An advantage of measuring CATHL4 as an inflammatory marker is that it can be detected in mastitic milk only and not in the milk of healthy animals [40,41]. The protein–protein interaction network analysis showed the well-characterized interaction between LTF and CATHL4, suggesting their dual protective role as AMP and key components of the innate arm of the immune system.

In the current study, peptidoglycan recognition protein-1 (PGLYRP1) showed the highest increase (eightfold) of all differentially abundant proteins in mastitic milk. PGLYRP1 is a conserved pattern recognition molecule, found mainly in polymorphonuclear leukocyte granules, that recognizes bacterial peptidoglycan and is bactericidal by way of inhibiting peptidoglycan synthesis [15,42]. LPO is known to exert antimicrobial effects and was previously reported as a biomarker of mastitis due to its role as an innate immune effector molecule. However, LPO was low-abundant in the current analysis of mastitic milk which may suggest that levels are decreased or limited by the low oxygen tension in the milk, as reported in previous studies of IMI with *S. uberis* and *S. aureus* [11,43,44].

Histone proteins (H4 and H2A) were detected in higher abundance in mastitic milk in the current analysis, which might reflect their well-characterized antimicrobial functions shown in earlier studies [45]. Indeed, a recent study revealed that histone proteins are one of the main components of neutrophil extracellular traps (NETs) produced in milk during mastitis [16,46,47]. NETs are considered to be a unique mechanism adopted by the neutrophils to limit and kill the invading bacteria through the production of a web of DNA, histones, and other antimicrobial proteins to trap and destroy bacteria [48,49]. The abundance of histone proteins in the mastitic whey in our analysis could be possibly due to their release as a result of cell lysis as well as NET disintegration [16].

The magnitude and the pattern/quantitative trends of the proteins present in mastitic milk compared to milk from healthy animals reflect the ability of *S. aureus* to modulate the host response as reported in other studies [16,20,50]. However, the complex nature and the high dynamic range of the milk proteome, the fractionation of whey proteins during sample preparation, and the dynamic exclusion of casein during MS analysis limited the number of low-abundant proteins that were identified. Additionally, the inherent biological variability of the immune response in infected cows during naturally occurring mastitis contributed to the inconsistent abundance of the identified proteins. However, taken together, our data suggest that high-abundance of host defense-related proteins represents modulation of the milk proteome during IMI with *S. aureus*. Quantitative proteomics using label-free analysis enabled the detection of proteomic signatures that reflect the host response in bovine subclinical mastitis and provided a comprehensive identification of proteins of low abundance in a broad molecular weight range that is typically missed with conventional gel-based approaches. Moreover, the bioinformatics analysis added an additional layer of information by mapping the identified proteins to significantly over-represented GO categories and by identifying protein–protein interactions that provide insight into the possible connections between the biological functions of the high-abundance proteins.

## 4. Methods

### 4.1. Sample Collection

Quarter milk samples used in this study were collected from 11 dairy cows from the Elora dairy research station at the University of Guelph. The cows were selected from the same herd and breed, were in mid to late lactation, and ranged in age from 4 to 8 years to avoid the effect of these confounding factors on the results. The criteria for selection of the cows were based on the results of clinical examination (absence of systemic and local signs) to exclude clinical mastitis. Cows in the control group had no previous history of clinical mastitis, had a low Somatic Cell Count (SCC), and had negative cultures for *S. aureus* in the two months preceding the study, whereas cows in the mastitic group had a previous history of subclinical mastitis, i.e., elevated SCC and positive bacterial culture but no systemic or localized signs of mastitis and no history of major systemic diseases. The use of all animals in this study was according to the guidelines of the Animal Care and Use Committee of the University of Guelph. (AUP # 1424, Approved time line: Monday, 30 April 2012 to Monday, 11 July 2016). All milk samples were visibly normal in gross appearance. Quarter milk samples (30 mL) were collected aseptically and brought immediately to the laboratory. Based on the Somatic Cell Count (SCC) and bacterial culture results, the samples were divided into two groups—a control group and a group with subclinical mastitis. Milk samples in the control group (*n* = 5) were microbiologically negative for *S. aureus* and had a SCC of less than 2 × 10^5^ cells/mL and the cows had no previous history of clinical mastitis in the two months preceding the study. Milk samples in the mastitic group (*n* = 6) were positive for *S. aureus* culture and had a high SCC of more than 2 × 10^5^ cells/mL. Milk samples for proteomic analysis were immediately mixed with a protease inhibitor cocktail (Roche Diagnostics GmbH, Mannheim, Germany), to minimize proteolysis and kept at −80 °C until further analysis. Microbiological analysis and SCC were performed within 24 h of collecting the samples.

### 4.2. SCC and Microbiological Examination

SCC was determined using a commercial automated cell counter (DeLaval Cell Counter DCC, Tumba, Sweden). The bacteriological analysis was performed according to the standard procedures of the National Mastitis Council [51]. Briefly, a loopful of each quarter milk sample was plated on Columbia Blood agar (CBA) supplemented with 5% sheep red blood cells, incubated at 37 °C, and examined at 24 h and 48 h for evidence of bacterial growth. *S. aureus* bacteria were identified by culture characteristic on selective media, gram-staining, and biochemical reaction [52]. The results of microbiological examination and SCC of both control and subclinical mastitic groups are shown in a supplemental table (Table S3). Bacterial cultures of milk samples from cows with subclinical mastitis infected with *S. aureus* showed typical morphological and biochemical properties of *S. aureus* including large, creamy hemolytic colonies on CBA, positive coagulase production within 18 h, and sugar–alcohol fermentation.

### 4.3. Separation of Whey Proteins

Whole milk samples were firstly centrifuged at 3000× *g* at 4 °C for 30 min to skim the milk and the fat layer was removed using a spatula. The remaining clear portion was transferred to an ultracentrifuge tube (Beckman Coulter Thickwall polycarbonate, Mississauga, ON, Canada) and centrifuged again in a Beckman Coulter benchtop ultracentrifuge (model LXL-TB-015BD) with a Swinging-Bucket Rotor (SW 32.1) at a speed of 45,000× *g* at 4 °C for 30 min. Casein micelles were pelleted in the sediment and the translucent supernatant forming the whey fraction was aliquoted into Eppendorf tubes and stored at −80 °C for further analysis. The total protein concentration was assessed in the whey fractions using the bicinchoninic acid assay (BCA) Protein Assay (BCA Protein Assay kit, Pierce™, ThermoFischer Scientific, Markham, ON, Canada).

### 4.4. In-Solution Trypsin Digestion of Whey Proteins

A quantity of 10 µg of whey protein in control and mastitic samples was dissolved in 20 µL of denaturation buffer (8 M urea, 50 mM Tris-HCl (pH 8). The following steps were carried out with incubation at room temperature and gentle shaking. Reduction of disulfide bonds was done by adding 0.2 µL of 5 mM dithiothreitol in 50 mM of ammonium bicarbonate (pH 7.8) and incubated at 37 °C for 1 h. For the alkylation step, iodoacetamide (0.4 µL) was added to a final concentration of 15 mM and incubated for an additional 30 min in the dark at room temperature. The reaction was diluted with three volumes of 50 mM Tris-HCl (pH 8) or 50 mM ammonium bicarbonate (pH 7.8) to allow for trypsin digestion. Finally, trypsin (Promega, product no. V5280) was added and incubated with the protein extracts overnight at 37 °C.

### 4.5. Liquid Chromatography and Tandem Mass Spectrometry (LC-MS/MS)

Following trypsin digestion, peptides were separated by on-line reverse-phase high-pressure liquid chromatography and mass spectrometry (LC-MS) using a LTQ-Orbitrap Elite (linear ion trap-orbitrap) hybrid analyzer outfitted with a nanospray source and EASY-SPRAY 1200 split free nano-LC system (ThermoFischer Scientific). A 50 cm PepMap RSLC Easy-Spray column filled with 2 µm C18 beads was used in the HPLC (ThermoFischer Scientific). The resultant peptides were loaded at a pressure of 800 BAR and eluted over 0–120 min at a rate of 250 nL/min using a 0–35% acetonitrile gradient in 0.1% formic acid. In the mass spectrometer, one full MS Scan (400–1500 *m*/*z*) was performed in the Orbitrap Elite with an automatic gain control (AGC) of 500,000, max ion time of 200 ms, and one microscan, at a resolution of 240,000. Ten data-dependent MS/MS scans were performed in the linear ion trap using the ten most intense ions at 35% normalized collision energy. MS and MS/MS scans were done in parallel. For MS/MS scans, the AGC was 10,000 with a maximum ion injection time of 100 ms. A minimum ion intensity of 1000 was required to trigger the MS/MS scan. Dynamic exclusion was applied with a maximum exclusion list of 500, with one repeat count and a repeat duration of 8 s and exclusion duration of 30 s.

### 4.6. Quantitative Proteomic Data Analysis

#### 4.6.1. Protein Identification and Label-Free Quantification

The raw MS/MS data from all samples were imported into a freely available computational proteomics platform, MaxQuant (version. 1.3.0.5, Max-Planck-Institute of Biochemistry, Martinsried, Germany), for label-free relative quantification analysis. Proteins were identified using *Bos taurus* protein reference proteomes (UniProt Proteome ID:UP000009136; last updated 10 May 2015) downloaded from the UniProt Knowledgebase and imported into the MaxQuant-integrated Andromeda search engine. Precursor mass accuracy of 7 ppm and MS/MS accuracy of 0.5 Da were performed during the main search. For identification and quantification, variable modification methionine oxidation and N-terminal acetylation and fixed modification cysteine carbamidomethylation were set for the search and enzyme specificity was set for trypsin where a maximum of two missed cleavages of trypsin was allowed for searching. The false discovery rate (FDR) for peptide–spectrum match and protein identification was set to 1% and was determined using the reversed peptide sequences (target–decoy–search strategy) to account for any false matches. At least one unique or “razor” peptide was required for protein identification. The frequently observed contaminants were removed after being compared to the *Bos taurus* reference proteome and MaxQuant contaminant list, where proteins were assigned to *Bos taurus* such as keratin and serum albumin protein if they were of bovine origin. Following protein identification, the intensity for each identified protein was calculated using peptide signal intensities (MS1). Retention time alignment, label-free quantification, and MaxLFQ normalization were done as described in the MaxLFQ label-free quantification method [53]. The (“match-between-runs”) feature in MaxQuant was enabled to run the identification transfer protocol within the experimental replicates to extract the quantification information across the replicates [53,54].

#### 4.6.2. Statistical Analysis

Perseus version 1.4.1.3 (Max-Planck-Institute of Biochemistry, Martinsried, Germany), the freely available software, was used to perform the statistical analysis wherein the LFQ protein intensities from the MaxQuant analysis were imported and transformed to a logarithmic scale with base 2. To obtain the quantitative data for all of the peptides in the samples, peak intensities from the whole set of measurements were compared [53]. The missing values were replaced with 20—the value of the lowest intensity—to compensate for the low signals of the low-abundant proteins. The statistical significance analysis and quantification were performed by two-way Student’s *t*-test using Perseus software. All proteins with a fold-change of at least 1.5 and FDR-adjusted (*p* < 0.05) were considered to be differentially abundant between the experimental groups. FDR-adjusted (*p* < 0.05) was corrected using the Benjamini–Hochberg multiple correction method. To further improve visualization and interpretation of the differentially abundant proteins, a Heatmap was generated using Perseus software.

#### 4.6.3. Gene Ontology (GO) Enrichment Analysis

To investigate the biological and potential clinical value of the identified differentially abundant proteins, further analysis was performed using enrichment and functional annotation analysis. Proteins were submitted to the Database for Annotation, Visualization, and Integrated Discovery (DAVID) v6.8 software with an updated knowledge base (available online: http://david.abcc.ncifcrf.gov) [55].

#### 4.6.4. Pathway and Network Analysis

To evaluate the integration of differentially abundant proteins through direct (physical) or indirect (functional) associations, we searched the STRING database for protein–protein interactions networks using the STRING v10.5 web-tool (Search Tool for the Retrieval of Interacting Genes/Proteins, (v10.5, EMBL, Heidelberg, Germany) [56]. The PPI enrichment *p*-value is 0.000273 with the application of a Fisher’s exact test followed by a correction for multiple testing. Each PPI in the STRING database is assigned a confidence score that ranges between 0 and 1. Also, we further identified and analyzed the enriched canonical pathways using ingenuity pathway analysis (IPA) software (v6.0, QIAGEN, Redwood City, CA, USA) [57].

The mass spectrometry proteomics data have been deposited to the ProteomeXchange Consortium via the PRIDE (available online: http://proteomecentral.proteomexchange.org) [58] partner repository with the dataset identifier *PXD007516*.

## 5. Conclusions

The study identified 90 proteins, 25 of which were differentially abundant between healthy and mastitic dairy cows. The functional analyses of these proteins showed strong links to host defense functions including pathogen-recognition, direct antimicrobial function, and the acute-phase response. The potential utility of these proteins for developing a diagnostic tool for subclinical mastitis will require further research and validation. Moreover, the comprehensive clinical, bacteriological, proteomic, and bioinformatics data presented in this study reinforces the data that are already available in the literature for comparison and validation purposes.

## Figures and Tables

**Table 1:** Proteins significantly high-abundant or low-abundant by the presence of *Staphylococcus aureus* identified by LC-MS/MS analysis in whey milk samples when compared to the control samples.

UniProt ID	Protein Name	Log2 Fold Change	*p*-Value	Unique Peptides Count
Q8SPP7	Peptidoglycan recognition protein 1	7.92 ^(+)^	0.000	10
Q2TBU0	Haptoglobin	6.45	0.002	17
Q3ZBX9	Histone H2A.J	5.47	0.000	1
P63258	Actin, cytoplasmic 2	5.46	0.002	9
E1B6Z6	Neutrophil gelatinase-associated lipocalin	5.29	0.001	13
P10096	Glyceraldehyde-3-phosphate dehydrogenase	5.17	0.003	8
Q1JPB0	Leukocyte elastase inhibitor (Serpin B1)	4.31	0.003	7
P33046	Cathelicidin-4 (Indolicidin)	4.29	0.002	4
G3X807	Histone H4	4.09	0.003	6
F1MYX5	Uncharacterized protein	4.08	0.002	13
P30922	Chitinase-3-like protein 1	3.97	0.01	16
G3MXB5	Uncharacterized protein	2.70	0.002	5
Q2TBI0	Lipopolysaccharide-binding protein	2.69	0.014	12
P07688	Cathepsin B	2.31	0.011	9
P24627	Lactotransferrin (Lactoferrin)	2.28	0.001	61
P79345	Epididymal secretory protein E1	−0.70 ^(−)^	0.013	9
P08037	Beta-1,4-galactosyltransferase 1	−1.08	0.011	12
Q9TUM6	Perilipin-2 (Adipophilin)	−1.55	0.006	18
P11151	Lipoprotein lipase	−1.57	0.007	13
Q95122	Monocyte differentiation antigen CD14	−1.70	0.00	9
Q0P569	Nucleobindin-1	−1.84	0.014	15
P80025	Lactoperoxidase	−1.93	0.001	31
G5E5H7	Uncharacterized protein	−2.18	0.009	3
Q4GZT4	ATP-binding cassette sub-family G member 2	−2.33	0.004	12
Q9XSG3	Isocitrate dehydrogenase	−2.92	0.013	10

(^+^) high-abundant proteins and (^−^) low-abundant proteins were present at significantly higher or lower levels, respectively, in mastitic cows compared with the healthy control cows.

**Table 2:** Biological processes (BP) that were significantly enriched in differentially abundant proteins during *S. aureus* mastitis.

GO Terms (Biological Processes) *	Gene Name	*p*-Value
GO:0042742~defense response to bacterium	*Hp*, *LPO*, *CATHL4*	0.0066
GO:0031640~killing of cells of other organism	*PGLYRP1*, *CATHL4*	0.0076
GO:0071223~cellular response to lipoteichoic acid	*LBP*, *CD14*, *PGLYRP1*	0.0091
GO:0034145~positive regulation of Toll-like receptor 4 signaling pathway	*LTF*, *LBP*	0.0136
GO:0006953~acute-phase response	*Hp*, *LBP*	0.027
GO:0019731~antibacterial humoral response	*LTF*, *LPO*	0.0314
GO:0031663~lipopolysaccharide-mediated signaling pathway	*LBP*, *CD14*	0.0417
GO:0045087~innate immune response	*LBP*, *CD14*, *PGLYRP1*	0.0459
GO:0098869~cellular oxidant detoxification	*Hp*, *LPO*	0.0461

* Gene ontology (GO) biological processes (BP) terms when direct option was used in the Database for Annotation, Visualization, and Integrated Discovery (DAVID) are indicated to avoid the redundancy of the terms enriched at *p* < 0.05 and 5% false discovery rate (FDR). LBP = Lipopolysaccharide-binding protein, CD14 = Monocyte differentiation antigen, LTF = Lactotransferrin, CATHL4 = Cathelicidin-4, Hp = Haptoglobin, (PGLYRP1) = Peptidoglycan recognition protein 1, LPO = Lactoperoxidase.

## Supplementary Materials

Supplementary materials can be found at www.mdpi.com/1422-0067/19/1/78/s1.
